# Development of Biocomposite Polymeric Systems Loaded with Antibacterial Nanoparticles for the Coating of Polypropylene Biomaterials

**DOI:** 10.3390/polym12081829

**Published:** 2020-08-15

**Authors:** Mar Fernández-Gutiérrez, Bárbara Pérez-Köhler, Selma Benito-Martínez, Francisca García-Moreno, Gemma Pascual, Luis García-Fernández, María Rosa Aguilar, Blanca Vázquez-Lasa, Juan Manuel Bellón

**Affiliations:** 1 Institute of Polymer Science and Technology, Spanish National Research Council (ICTP-CSIC), 28006 Madrid, Spain; mar.fernandez.gutierrez@csic.es (M.F.-G.); luis.garcia@csic.es (L.G.-F.); mraguilar@ictp.csic.es (M.R.A.); bvazquez@ictp.csic.es (B.V.-L.); 2 Biomedical Networking Research Centre on Bioengineering, Biomaterials and Nanomedicine (CIBER-BBN), 28029 Madrid, Spain; selma.benito@uah.es (S.B.-M.); francisca.garciam@uah.es (F.G.-M.); juanm.bellon@uah.es (J.M.B.); 3 Department of Medicine and Medical Specialties, University of Alcalá, 28805 Madrid, Spain; 4 Ramón y Cajal Health Research Institute (IRYCIS), 28034 Madrid, Spain; 5 Department of Surgery, Medical and Social Sciences, University of Alcalá, 28805 Madrid, Spain

**Keywords:** biocomposite, chitosan, chlorhexidine, coating, hernia, mesh infection, nanoparticles, PLGA, polypropylene, rifampicin

## Abstract

The development of a biocomposite polymeric system for the antibacterial coating of polypropylene mesh materials for hernia repair is reported. Coatings were constituted by a film of chitosan containing randomly dispersed poly(d,l-lactide-*co*-glycolide) (PLGA) nanoparticles loaded with chlorhexidine or rifampicin. The chlorhexidine-loaded system exhibited a burst release during the first day reaching the release of the loaded drug in three or four days, whereas rifampicin was gradually released for at least 11 days. Both antibacterial coated meshes were highly active against *Staphylococcus aureus* and *Staphylococcus epidermidis* (10^6^ CFU/mL), displaying zones of inhibition that lasted for 7 days (chlorhexidine) or 14 days (rifampicin). Apparently, both systems inhibited bacterial growth in the surrounding environment, as well as avoided bacterial adhesion to the mesh surface. These polymeric coatings loaded with biodegradable nanoparticles containing antimicrobials effectively precluded bacterial colonization of the biomaterial. Both biocomposites showed adequate performance and thus could have potential application in the design of antimicrobial coatings for the prophylactic coating of polypropylene materials for hernia repair.

## 1. Introduction

In hernia repair, the implantation of biomaterials constitutes the main surgical strategy aimed at reinforcing the abdominal wall, providing a tension-free repair that restores the biological, biomechanical, and functional properties of the damaged tissue [1]. Among the numerous advantages afforded by these devices, the use of hernia mesh materials allows for the reduction of recurrence rates and recovery period [2]. Nevertheless, mesh implantation is not exempt from drawbacks, being that infection is one of the most severe postoperative complications having a big impact on patients and the healthcare system [3]. Many factors influence the incidence of mesh-related infection, such as the type of hernia pathology, the surgical procedure carried out, the prostheses implanted, and the patient co-morbidities among the most relevant [4].

Typically, biomaterial-related infections are triggered when bacteria invade the wound and adhere to the surface of the implanted mesh and surrounding tissues. Once adhered, bacteria proliferate and interact with others, forming clusters that colonize the implant [5]. Many of these bacteria secrete an extracellular polysaccharide matrix that covers the cluster and strengthen their adhesion to the surface, establishing a biofilm [6]. Protected by this barrier, bacteria are strongly attached to the substrate and exert high resistance to the action of antibiotics and host defense cells [7], events that make biofilm-based infections extremely difficult to treat. Most of these infections, either involving biofilms or not, are caused by *Staphylococcus aureus* and *S. epidermidis* [8].

Considering that bacterial adhesion to the implant surface plays a key role in the pathogenesis of mesh-related infections, there is a growing interest in developing novel surgical meshes endowed with antibacterial properties. In this sense, coating meshes with drug-loaded polymeric systems is currently one of the main approaches being evaluated by researchers [1,9]. In recent years, numerous laboratory and preclinical studies demonstrated that antimicrobial coatings could reduce mesh-related infections by inhibiting bacterial adhesion, implant colonization, and biofilm formation [3,10,11].

Different antimicrobials have been used to endow hernia mesh materials with antibacterial properties. Silver metal [12] or antibiotics such as gentamicin [13], vancomycin [14], and rifampicin–minocycline combinations [15] are among the most widely used drugs for hernia mesh coating. Although exerting a potent antibacterial activity, it is widely known that antibiotics carry the risk of provoking bacterial resistances, thus, the prophylactic use of these biocides should be reduced to a minimum. Antiseptic agents emerge as a plausible alternative to antibiotics for these purposes. From the wide variety of antiseptics available, some have been used to coat mesh materials with promising results, being a potential tool for developing antimicrobial devices. Such is the case of broad-spectrum antiseptics like triclosan [16] and chlorhexidine [17] both of which are currently being applied in the manufacturing of antimicrobial surgical devices for clinical use [11].

The simplest way to coat a mesh is via dipping or soaking the device in aqueous solutions containing the target antimicrobial immediately before its implantation. Although this strategy is often applied in human clinics [18], it fails to provide controlled levels of the drug at the surgical site. This limitation can be alleviated if the mesh is coated with a polymeric compound loaded with antimicrobials. Theoretically, antibacterial mesh polymeric coatings must provide a local, sustained, and slow drug release at the implant site, neither producing local nor systemic detrimental effects due to the diffusion of the antimicrobial [14]. Furthermore, coatings must be non-toxic and biocompatible, ensuring adequate mesh integration into the host tissue without compromising the biomechanical properties of the implant [19]. Over the last decade, many polymeric systems to be used as drug carriers were developed and applied for the prophylactic coating of hernia repair materials, with promising outcomes [11,20]. From this plethora of candidates, the polymeric systems loaded with drug releasing nanoparticles represent one of the most effective approaches to endow these devices with antibacterial activity [5].

In the present study, we developed a novel antimicrobial polymeric coating loaded with biodegradable nanoparticles containing chlorhexidine or rifampicin for hernia mesh materials. The nanoparticles were loaded with two different antimicrobials, chlorhexidine or rifampicin, as candidates for antiseptic and antibiotic agents, respectively. The former (C_22_H_30_Cl_2_N_10_) is a cationic biguanide antiseptic widely used as a preoperative skin disinfectant [21]. The latter (C_43_H_58_N_4_O_12_) is considered one of the most potent semisynthetic antibiotics to treat bacterial infections [22]. Both drugs were selected due to their antimicrobial activity as well as their promising application with biomedical devices. The morphology, drug release profile, cytocompatibility, and antimicrobial performance of coated polypropylene meshes was evaluated in vitro using a model of bacterial mesh infection caused by *S. aureus* and *S. epidermidis*.

## 2. Materials and Methods

### 2.1. Chemicals

Commercial reagents and antimicrobials used for the coating preparation were: chitosan (medium molecular weight, deacetylation degree 75%; Sigma-Aldrich, St. Louis, MO, USA); dichloromethane, DCM (Sigma-Aldrich); poly(d,l-lactide-*co*-glycolide), PLGA, Resomer RG503 (50/50; Evonik, Essen, Germany); chlorhexidine, CHX (Sigma-Aldrich); and rifampicin, RIF (Sigma-Aldrich).

### 2.2. Coating Preparation

The elaboration of the polymeric coatings was conducted at room temperature. For the establishment of the unloaded polymer, chitosan was dissolved in 1% acetic acid with continuous magnetic stirring for 24 h at a concentration of 1.0% w/v. Then, PLGA was dissolved in DCM (25% w/w vs. chitosan) and subsequently loaded into a 1 mL syringe. This PLGA solution was added to the chitosan solution drop by drop using a 23-gauge needle, and the obtained chitosan–PLGA dispersion was stirred for 12 h for evaporation of the organic solvent DCM. In the case of the drug-loaded polymers, CHX (2% w/w vs. chitosan) or RIF (5% w/w vs. chitosan) were dissolved in DCM and the mixture with the solution of PLGA was added drop by drop slowly, to the chitosan aqueous solution under stirring for 12 h. In this time period, the organic solvent DCM evaporated with the subsequent formation of solid nanoparticles containing the biodegradable polymer and the corresponding antiseptic or antibiotic drug as a nanodispersion in the original aqueous solution of chitosan, which was applied for the homogeneous coating of the commercial mesh. According to the amount ratio of PLGA and drug, CHX nanoparticles contained 10% (w/w) of the drug, whereas those prepared with RIF contained 15% (w/w) of the bioactive drug.

### 2.3. Mesh Coating and Study Groups

A lightweight, monofilament, polypropylene (PP) mesh material was used (Optilene Mesh Elastic; B. Braun, Melsungen, Germany). Under sterile conditions, the mesh was cut into 1 cm^2^ squares that were subsequently coated with the different polymeric compounds, by means of the casting technique. Using a Pasteur pipette, 300 µL of the corresponding compound were dropped onto each mesh, and coated samples were dried at room temperature for 24 h. The coated meshes were sterilized with ethylene oxide at 40 °C for 20 h. Four study groups were established:

- Control (uncoated PP).
- Pol (PP coated with unloaded polymer).
- Pol-CHX (PP coated with CHX-loaded polymer).
- Pol-RIF (PP coated with RIF-loaded polymer).

### 2.4. Visualization of the PLGA-Nanoparticles Loaded Biocomposites

The size and distribution of the nanoparticles from the original dispersion of nanoparticles in the solution of chitosan was analyzed under scanning electron microscopy (SEM), via the deposition of a few drops on the sample holder of the microscope. Moreover, samples from the different study groups (*n* = 3 each) were used to evaluate the homogeneity and distribution of the polymeric coating. The meshes were dehydrated in an increasing graded ethanol series (30%, 50%, 70%, 90%, and 100%, incubation for 15 min each), desiccated in a Polaron CPD7501 (Fisons Instruments, Ipswich, UK), gold-palladium coated, and visualized in a Zeiss DSM950 SEM (Carl Zeiss, Oberkochen, Germany).

### 2.5. Drug Release

Samples from the different study groups (*n* = 3 each) were transferred to vials containing 5 mL of phosphate buffer saline (PBS, pH 7.4) at 37 °C. At different time intervals in a 14-day period, aliquots were taken for further analysis, and the vials were refilled with the same volume of fresh PBS. The collected aliquots were mixed with the mobile phase (1:1), consisting of a solution of acetonitrile: water (20:80), and filtered. The drug release was analyzed by means of high-performance liquid chromatography (HPLC) using a Shimadzu SL-200 apparatus (Shimadzu Scientific Instruments Inc., Columbia, SC, USA) with a Kromaphase C18 column (250 mm × 4.6 mm) and eluting rate of 1 mL/min, using a UV detector at 254 and 280 nm for CHX and RIF, respectively. Calibration curves using a free drug were previously obtained with good correlation values (r = 0.9887 for CHX and r = 0.9668 for RIF). Three measurements were taken for each sample. Results were plotted in a graph representing the cumulative percentage drug release over time.

### 2.6. Cell Viability

A cell viability assay was conducted to determine the in vitro biocompatibility of the designed mesh coatings, by means of the alamarBlue test. The alamarBlue is a colorimetric reagent (active ingredient: resazurin) used to measure cell proliferation and cytotoxicity of eukaryotic cells or bacteria, following exposure to chemicals or drugs; cells exerting a proliferative status will reduce this reagent (converting resazurin to resorufin), which provokes a colorimetric change in the culture medium measurable by spectrophotometry. For this assay, rabbit skin fibroblasts were used, which were isolated by the explant method, as described elsewhere [17]. Biopsies were harvested from the dermis tissue of 3 male New Zealand White rabbits belonging to another study, (experimental protocol approved by the Committee on the Ethics of Animal Experiments of the University of Alcalá, reference PROEX 045/18). Cells were cultured in a controlled humid atmosphere (37 °C, 5% CO_2_) in Dulbecco’s modified Eagle medium (DMEM) containing 10% fetal bovine serum (FBS) plus 1% antibiotic–antimycotic cocktail for cell culture (Life Technologies, Carlsbad, CA, USA), and visualized in a Zeiss Axiovert 40C phase-contrast inverted microscope (Carl Zeiss, Oberkochen, Germany). Cells were grown in 6-well plates (2.5 × 10^5^ cells per well in 2 mL of DMEM) and incubated overnight under the conditions described above. Next, the medium was renewed, and a mesh fragment of the corresponding study group was transferred to each well (*n* = 9 per group). Following a 24 h incubation period under the same conditions, the medium was discarded, wells were washed in Hank’s balanced salt solution (Life Technologies) and 2 mL of FBS-free DMEM supplemented with 10% alamarBlue viability reagent (Bio-Rad, Hercules, CA, USA) was added. After 5 h of incubation at 37 °C, several 100 µL aliquots were collected from each well to read absorbance (OD570 and OD600) in an iMark microplate absorbance reader (Bio-Rad, Hercules, CA, USA). Data were analyzed using software provided by the manufacturer. Results were expressed as the mean percentage cell viability.

### 2.7. Antibacterial Performance of the Biocomposites

To determine the performance of the antibacterial coatings, fragments from the different study groups were inoculated with *S. aureus* (Sa) ATCC25923 and *S. epidermidis* (Se) ATCC35984, purchased from the Spanish Type Culture Collection (Valencia, Spain). In all the experiments carried out, the inoculating dose was 10^6^ CFU/mL. Bacterial suspensions were prepared immediately before starting the procedures. To avoid cross-contamination, all the assays described below were developed in an independent manner for Sa and Se.

#### 2.7.1. Elaboration of the Bacterial Suspensions

Bacteria were thawed, spread onto lysogeny broth (LB) agar plates (Biomérieux, Marcy l’Etoile, France) and incubated for 24 h at 37 °C. A colony was transferred to 25 mL of LB broth. Following overnight incubation at 37 °C, the culture was diluted in sterile 0.9% saline to prepare the target inoculum (1.25–1.5 × 10^6^ CFU/mL), by spectrophotometry (OD600). The number of viable CFU in each inoculum was determined by the spot plaque method.

#### 2.7.2. Sequential Agar Well Diffusion Test

A variant of the agar well diffusion test was carried out to determine the capacity of the drug-loaded mesh coatings to sequentially create zones of inhibition (ZOIs) over a 14-day period of study. Briefly, several LB agar plates were inoculated with 1 mL of the corresponding bacterial suspension. Then, samples from the different study groups (*n* = 6 per group and strain) were individually placed onto the agar and incubated at 37 °C for 24 h. Following incubation, all the plates were photographed for further ZOIs measurement. Those samples exhibiting ZOIs were transferred to freshly inoculated LB plates and incubated again under the same conditions. For each sample, this process was repeated every 24 h until no ZOI was observed in the agar, or up to day 14, if the ZOIs were still being developed. Once the assay was finished, the pictures previously taken were used to quantify the ZOIs amplitude at every 24-h interval. For this quantification, two perpendicular diameters of the ZOIs were measured, using the ImageJ software (National Institutes of Health, Bethesda, MD, USA). Results were plotted in a graph representing the mean ZOI (mm) of the different study groups over time.

#### 2.7.3. Bacterial Adhesion to the Meshes

The ability of the different coatings to avoid bacterial adhesion to the mesh surface was assessed by sonication. Samples (*n* = 6 per group per strain) were individually immersed in 3 mL of LB broth, in 6-well plates. Then, each well was inoculated with 1 mL of the corresponding inoculum, and plates were incubated at 37 °C for 24 h. Following incubation, the meshes were carefully washed in 1 mL of sterile 0.9% saline to remove the non-adhered bacteria and subsequently transferred to Falcon tubes containing 10 mL of peptone water. The tubes were sonicated for 10 min at 40 KHz in a Bransonic 3800-CPXH ultrasonic bath (Branson Ultrasonics, Danbury, CT, USA). The supernatant in each tube was vortexed for 1 min, serially diluted in peptone water (6 tenfold dilutions), and 100 µL of the supernatant plus serial dilutions were spread on LB agar plates. Following a 24-h incubation at 37 °C, the plates were counted to quantify the bacterial adhesion to the surface of the meshes. Results were expressed as the mean bacterial yields per study group. 

#### 2.7.4. Turbidimetric Determination of the Bacterial Growth

Spectrophotometric assays were carried out to determine whether the presence of the coated meshes inhibited the bacterial growth in culture. As previously described, samples from the different groups (*n* = 6 per group per strain) were immersed in 3 mL of LB broth, inoculated with 1 mL of the corresponding bacteria and incubated 37 °C for 24 h. As blank, mesh samples immersed in 3 mL of LB broth plus 1 mL of sterile 0.9% saline were kept under the same conditions. Following incubation, the liquid was aspirated, vortexed, and measured by spectrophotometry (OD600) using an Ultrospec 3100 Pro spectrophotometer (Amersham Biosciences, Little Chalfont, UK). Results were expressed as the mean absorbance recorded for the different study groups. 

### 2.8. Statistical Analysis

The data collected in the different experiments were expressed as the mean ± standard error of the mean. Data were compared by a one-way analysis of variance (ANOVA) and Bonferroni as the post hoc test. All statistical tests were performed using GraphPad Prism 5.0 (GraphPad Software Inc., La Jolla, San Diego, CA, USA). Significance was set at *p* < 0.05.

## 3. Results

### 3.1. Bioactive Polymeric Film 

**Figure 1:**
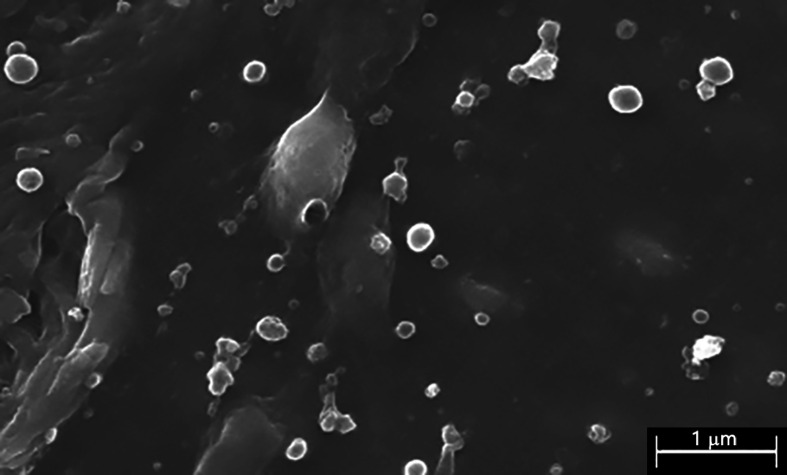
Scanning electron microscopy (SEM) micrograph of PLGA nanoparticles loaded with chlorhexidine, randomly distributed in a chitosan film (scale: 1 µm). The nanoparticles’ size ranges from 300 to 400 nm. Similar observations were recorded for the rifampicin-loaded polymer.

**Figure 2:**
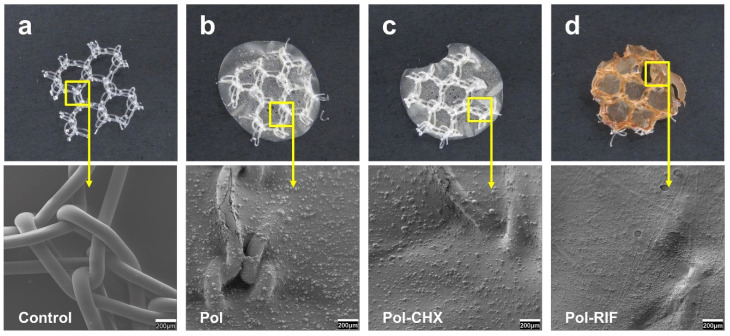
Macroscopic pictures and SEM micrographs (scales: 200 µm) of (**a**) nude control meshes, (**b**) meshes coated with the unloaded biocomposite (Pol), (**c**) meshes coated with chlorhexidine-loaded biocomposite (Pol-CHX), and (**d**) meshes coated with the rifampicin-loaded biocomposite (Pol-RIF).

The immiscibility of the organic solvent DCM with the diluted acetic acid solution in water provided an excellent tool for the preparation of a biocomposite system, formed by nanoparticles of PLGA loaded with either CHX or RIF dispersed in the diluted aqueous solution of chitosan. The chitosan solution containing the nanoparticles was a homogeneous dispersion of the particles formed after the evaporation of the volatile organic solvent DCM under the stirring of the dispersion of chitosan, thus avoiding the need of isolating such dispersion before the application of the biocomposite system to the mesh materials. Figure 1 shows a micrograph of a dry film of chitosan containing nanoparticles and it is easy to distinguish that the nanoparticles were homogenously and randomly distributed in all the films analyzed by SEM, with a PLGA-CHX nanoparticle size ranging from 300 to 400 nm on average. A similar result with the PLGA-RIF system was obtained. We observed that after the evaporation of the water and sterilization at 40 °C, the amount of residual DCM in the dry film was practically zero. The architecture of the different coatings with pure chitosan or chitosan charged with nanoparticles was visualized in SEM after the application to the commercial meshes (Figure 2). The uncoated mesh exhibited its characteristic architecture, consisting of a knitted PP monofilament that created large pores with a honeycomb-like structure. Once coated, the surface of this biomaterial turned different. The polymeric compound formed a thin layer that covered the mesh filaments and the pores in a continuous fashion.

### 3.2. Release Kinetics of CHX and RIF

**Figure 3:**
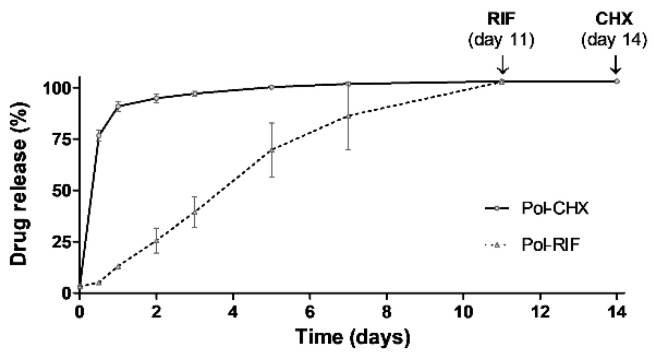
Release profile of CHX and RIF from the chitosan–PLGA nanoparticles polymeric coating over time, following its hydration in PBS (*n* = 3 each). Maximum cumulative release was observed after 11 and 14 days for RIF and CHX, respectively.

Results from the drug release studies revealed a different response between the two antimicrobials (Figure 3). The antiseptic drug CHX exhibited a moderate burst release during the first 12 h, followed by a gradual release that was extended for 14 days. However, the antibiotic RIF displayed a more gradual release profile, reaching the total release by day 11. No release was detected in the Pol group, revealing the absence of products of degradation generated by the polymeric coating.

### 3.3. Cell Compatibility of the Biocomposite

**Figure 4:**
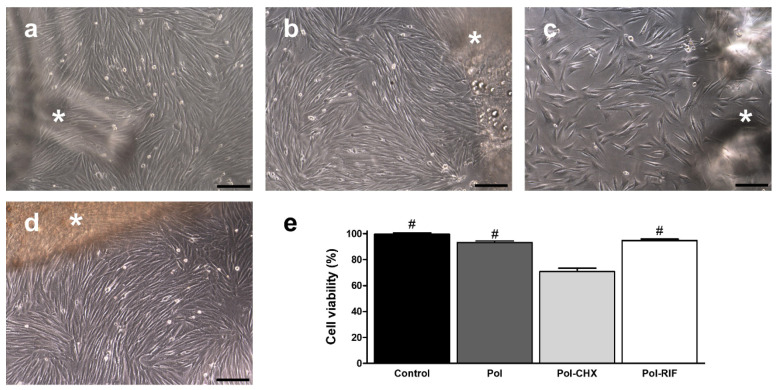
Light-microscopy micrographs (scales: 100 µm) of rabbit skin fibroblast cultured in the presence of a (**a**) control, (**b**) Pol, (**c**) Pol-CHX, or (**d**) Pol-RIF material. In each picture, the corresponding mesh is depicted (asterisks). The cells influenced by Pol-CHX coated meshes (**c**) exhibited slight morphological changes. (**e**) Analysis of cell viability after a 24-h exposure to the different materials (*n* = 9 each). Viability of fibroblasts decreased when cultured with Pol-CHX, compared to the rest of the study groups (#: *** *p* < 0.001).

Cultured fibroblasts exhibited a typical elongated shape and multilayered growth pattern, showing numerous cells undergoing mitosis. This morphology remained unchanged following exposition to the control, Pol and Pol-RIF meshes (Figure 4), keeping fibroblasts their proliferative status. However, cells influenced by Pol-CHX meshes turned stellate and a diminished proportion of mitotic figures was evidenced. Results from the alamarBlue assays revealed equivalent viability among cells cultured in the presence of either the uncoated (99.51% ± 1.10%), Pol (93.21% ± 1.13%), and Pol-RIF meshes (94.65% ± 1.21%), while fibroblasts exposed to Pol-CHX (70.74% ± 2.69%) showed a significantly lower percentage viability (*p* < 0.001). 

### 3.4. Antibacterial Performance of the Biocomposites

#### 3.4.1. Control of the Bacterial Load

The quantification of all the bacterial suspensions used yielded viable CFU values within the expected range. On average, bacterial load from the different inocula were 1.43 × 10^6^ CFU/mL and 1.41 × 10^6^ CFU/mL for Sa and Se, respectively.

#### 3.4.2. Antibacterial Activity over Time

**Figure 5:**
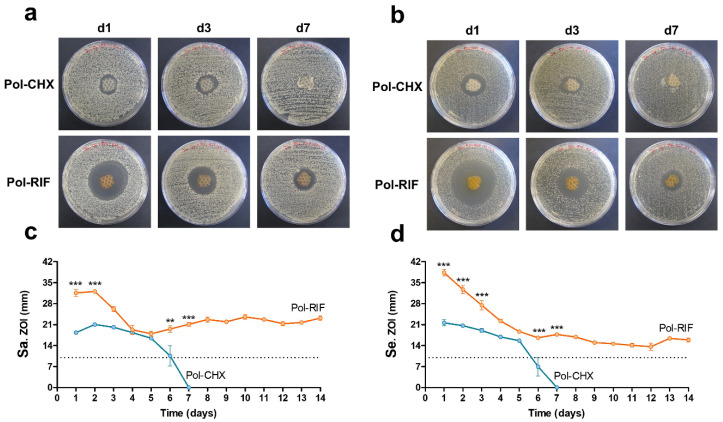
Antibacterial activity over time. Upper panel: Macroscopic pictures of the zones of inhibition (ZOIs) developed by Pol-CHX and Pol-RIF coated meshes at days 1, 3, and 7 of the initial inoculation with (**a**) Sa and (**b**) Se. Lower panel: Representation of the 24-h variation of the ZOIs amplitude of Pol-CHX and Pol-RIF coated meshes challenged with (**c**) Sa and (**d**) Se (** *p* < 0.01; *** *p* < 0.001). The length of the mesh fragments is depicted (dashed lines).

The sequential agar well diffusion test was aimed at determining the ability of the polymeric coatings to exert antibacterial activity over time (Figure 5). As expected, no ZOIs were developed by the control and Pol meshes, while a different response was observed with the antiseptic and the antibiotic-loaded coatings. Regardless of the bacteria inoculated, Pol-CHX meshes developed stable ZOIs that gradually decreased until full depletion following 7 days of the initial inoculation. By contrast, the ZOIs displayed by Pol-RIF were still stable by day 14. The statistical evaluation of the ZOIs amplitude was performed individually for each bacterial strain. When inoculated with Sa, ZOIs from the Pol-RIF meshes were wider than those from the Pol-CHX group, especially at days 1, 2 (*p* < 0.001), 6 (*p* < 0.01), and 7 (*p* < 0.01). Similar findings were collected in the Se-inoculated samples, with statistical relevance at days 1, 2, 3, 6, and 7 (*p* < 0.001). 

#### 3.4.3. Prevention of Bacterial Adhesion

The quantification of bacteria yielded from the different meshes following sonication (Table 1) revealed a strong adhesion to the surface of the control and Pol materials. For both Sa and Se, loads collected from the uncoated meshes were about 2-log smaller than the yields from the Pol materials (*p* < 0.01). Contrary to this, the antimicrobial coatings were highly effective; out of the total Pol-CHX and Pol-RIF meshes inoculated with either Sa or Se, only one sample belonging to the Pol-CHX group exhibited positive Se counts (1.10 × 10^3^ CFU), while the surface of the rest of fragments was free of bacteria. Furthermore, the bacterial load found in this sample was significantly lower than the yields from the control and Pol groups (*p* < 0.01).

#### 3.4.4. Inhibition of Bacterial Growth

**Figure 6:**
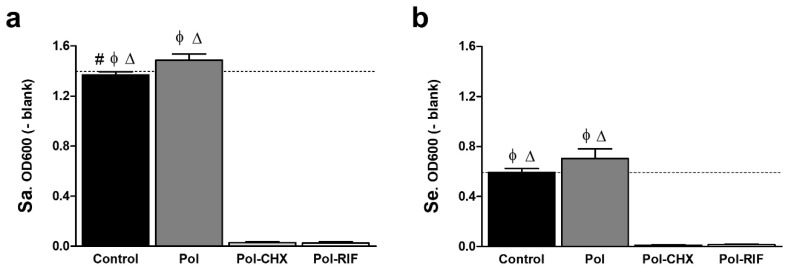
Absorbance (OD600) of (**a**) Sa and (**b**) Se cultures influenced by the different materials (*n* = 6 each). The absorbance of bacteria cultured without mesh is depicted (dashed lines). Regardless of the strain, no growth was recorded when bacteria were exposed to the antimicrobial biocomposites. Statistical significance was as follows: #: vs. Pol (* *p* < 0.05); ϕ: vs. Pol-CHX (*** *p* < 0.001); ∆: vs. Pol-RIF (*** *p* < 0.001).

Bacterial suspensions containing the different meshes were incubated for 24 h to determine whether the antimicrobial biocomposites did influence the growth pattern of Sa and Se, by means of a turbidimetric assay (Figure 6). Data collected from the absorbance recordings revealed a similar response of Sa and Se to the mesh coatings. For both strains, the absorbance slightly increased in those cultures containing Pol meshes compared to the control ones, being this increase statistically relevant for Sa (*p* < 0.05). In turn, and regardless of the bacterial strain, cultures containing Pol-CHX and Pol-RIF exhibited no turbidity and the values of absorbance significantly decreased in comparison with the rest of the groups (*p* < 0.001).

## 4. Discussion

In general surgery, abdominal wall hernia repair constitutes one of the most frequently developed procedures. According to a recent Cochrane review, approximately 20 million surgeries are being performed per year worldwide [23]. In those patients, bacterial mesh infections increase the morbidity and mortality rates, lengthen the hospital stay and raise the sanitary costs [24]. Hence, the clinical and social consequences of mesh infections are of major concern.

One of the biggest issues associated with this postoperative complication is that currently there are no gold-standard therapies for preventing mesh-related infections [25]. Preoperative surgical antibiotic prophylaxis is common practice, although it fails to avoid infections in many cases [26] and there is no clear consensus among surgeons regarding its administration [27]. In this milieu, the implantation of antimicrobial meshes constitutes a promising option to reduce the incidence of infection.

Antimicrobial prostheses can be developed following different procedures; the most attractive alternative consists of coating the mesh with bioactive polymeric systems. Ideally, these coatings must cover the surface uniformly, providing an adequate drug concentration at the implant site [28]. Moreover, the coating should be biodegradable, facilitating both infiltration of neoformed connective tissue across the mesh pores and mesh integration into the host tissue [14]. Finally, the drug diffusion from the polymer should be gradual and maintained for about 3–4 days [29,30] to keep bactericidal activity over time. The innovative chitosan–PLGA nanoparticles system designed in this study combines all these requisites, especially when loaded with the antibiotic; firstly, observations from the different experiments carried out revealed signs of polymer degradation, suggesting adequate biodegradability of the biocomposite. Secondly, not only is this coating homogeneously distributed throughout the mesh surface, but also allows for a sustained release of rifampicin for up to 11 days. Nevertheless, the meshes coated with the antiseptic-loaded polymer exhibited an initial burst release during the first 24 h. These findings can be related to the chemical nature of chlorhexidine; this molecule is positively charged and has high binding affinity, features that often hinder the dilution of this antiseptic in polymeric systems and drug carriers [31,32].

Together with the chemical composition, the biocide activity of the selected drug is of great importance when developing antibacterial coatings for biomedical devices. The two antimicrobials included in this study were chosen because of their broad-spectrum activity against Gram-positive and Gram-negative bacteria, as well as their potential usefulness to prevent biomaterial-related infection following hernia repair. Although both drugs are effective biocides, the antiseptic and the antibiotic used have different mechanisms of action. On one side, chlorhexidine interacts with the proteins of the bacterial cell wall, provoking the disruption of the cytoplasmic membrane and the leakage of several cytoplasmic components [33]. By comparison, rifampicin provokes genetic damage caused by a strong, specific inhibition of the bacterial DNA-dependent RNA polymerase that hinders the RNA transcription processes [34]. Observations from our study revealed that polymeric films containing these drugs killed bacteria during the first 24 h of exposure, suggesting a fast, almost immediate biocide performance of these biocomposites, regardless of their mode of action.

Given that mesh infection can arise days, weeks, or even months after surgery [25], the development of antibacterial coatings exerting sustained biocide activity over time would be highly advantageous. It has been suggested that implanted antimicrobial devices should keep activity for 2–3 weeks [30]. Consistent with this statement, our rifampicin-loaded biocomposite exhibits biocide activity for at least 2 weeks. Notwithstanding this, the activity of the coating containing chlorhexidine lasted for at about 1 week. Likewise, rifampicin-loaded materials fully inhibited bacterial adhesion to the mesh surface, while the chlorhexidine-loaded ones allowed mild adhesion of *S. epidermidis* in just one sample. Together, these results reveal a stronger effect and better long-term activity of the antibiotic compared to the antiseptic bioactive system. To the best of our knowledge, no previous data were reported comparing the effect of these two drugs relative to hernia mesh materials, except for another study recently carried out by us [35]. In alignment with this, a clinical trial on the use of antibacterial central venous catheters to reduce bloodstream infections revealed better performance of those devices containing rifampicin compared to others provided with chlorhexidine [36].

Biocompatibility of the polymeric system is another key feature to be considered when designing antimicrobial coatings. In a setting of infection, mesh integration can be hampered if bacteria win the so-called “race for the surface” and colonize the implant before the host cells [37]. Biocompatible devices enhance the interaction of host cells with the implant promoting tissue integration [30,38], thereby indirectly reducing the establishment of infections. Recently, PLGA nanoparticles modified with chitosan were reported as effective drug carriers given their great biocompatibility and sustained release [39]. Our findings align with these observations and show that the biocomposites, especially those provided with rifampicin, exert no detrimental effects on cultured cells.

Given that rifampicin specifically targets the bacterial DNA-dependent RNA polymerase, we confirmed that, at the concentration tested, this drug does not provoke any side effect on fibroblasts in terms of cell viability. However, cytotoxicity of chlorhexidine to eukaryotic cells is well known [33,40]. It was demonstrated that this antiseptic could reduce the growth pattern of cultured cells, as well as provoke morphological modifications on fibroblasts [41], which corroborates our cell viability results. The main reason for this effect is that chlorhexidine can also disrupt the cytoplasmic membrane of eukaryotic cells, even when used at very low concentrations [42]. Together, these findings suggest a potential limitation of this antiseptic for further biomedical applications. In previous studies, our group managed to reduce the toxicity of this antiseptic via incorporation into a quaternary-ammonium-based polymeric compound [17]. With the development of this chitosan–PLGA polymeric system we have optimized the cell response to chlorhexidine, producing a more efficient antimicrobial mesh coating that exerts reduced toxicity while keeping a proper antibacterial activity.

In summary, the results described in the present study demonstrate that a chitosan–PLGA polymeric system can be loaded with antimicrobials to develop a bioactive coating for hernia repair mesh materials. Although the antibiotic rifampicin exhibits an optimal effect, the incorporation of an antiseptic agent such as chlorhexidine also shows an adequate response. Both coatings exert a desirable drug release profile, cell compatibility, or antimicrobial activity. The development of in vivo preclinical studies would provide further data regarding the potential application of these antimicrobial biocomposites to reduce postoperative infections following hernia repair. These steps will be carried out in the next future.

## 5. Conclusions

Polypropylene mesh materials for hernia repair can be coated with a bioactive chitosan–PLGA nanoparticle polymeric system loaded with either an antibiotic (rifampicin) or an antiseptic (chlorhexidine). Both biocomposite coatings effectively inhibited the bacterial colonization of the mesh without compromising the viability of eukaryotic cells. Rifampicin exerted slightly better performance than chlorhexidine in terms of drug release kinetics, cell compatibility, and antibacterial activity over time. The prophylactic application of these coatings offers a potential strategy to protect meshes from bacterial adhesion following implantation.

## Figures and Tables

**Table 1:** Bacterial adhesion to the surface of the different materials. Results were expressed as mean, median, minimum, and maximum bacterial loads (recorded as colony forming units, CFU) yielded per study group and bacteria (*n* = 6 each). The percentage of samples yielding positive counts (%) is provided.

Bacteria	Value	Control	Pol	Pol-CHX	Pol-RIF
Sa	Mean (CFU)	1.09 × 10^7^	3.41 × 10^9^	0	0
	Median (CFU)	9.15 × 10^6^	2.54 × 10^9^	0	0
	Min. (CFU)	5.00 × 10^6^	2.24 × 10^8^	0	0
	Max. (CFU)	2.01 × 10^7^	1.23 × 10^10^	0	0
	Positive (%)	100 (6/6)	100 (6/6)	0 (0/6)	0 (0/6)
Se	Mean (CFU)	1.12 × 10^5^	7.08 × 10^7^	1.83 × 10^2^	0
	Median (CFU)	6.00 × 10^4^	6.70 × 10^7^	0	0
	Min. (CFU)	2.00 × 10^4^	1.98 × 10^7^	0	0
	Max. (CFU)	3.60 × 10^5^	1.49 × 10^8^	1.10 × 10^3^	0
	Positive (%)	100 (6/6)	100 (6/6)	16.67 (1/6)	0 (0/6)
